# Book Review: Brain, Mind and Consciousness

**DOI:** 10.3389/fpsyg.2021.675976

**Published:** 2021-05-14

**Authors:** Jing Zhang, Da Dong

**Affiliations:** ^1^School of Marxism, Institute of Philosophy, Hangzhou Dianzi University, Hangzhou, China; ^2^Department of Philosophy, Center for the Study of Language and Cognition, Zhejiang University, Hangzhou, China

**Keywords:** brain, mind, consciousness, mental activities, components of consciousness, unified theory of psychology and cognition

Until now, the problem of consciousness is famously open to diverse sorts of philosophical and scientific solutions. With the aid of neurotechnology, some researchers acknowledge that the science of consciousness is a “well-established field of empirical research” (Doerig et al., 2020, p. 1), while other people disagree. Nevertheless, almost nobody deems a promising approach to this problem without unraveling the crux of (human) brain (for recent discussions, see, e.g., Gennaro, 2018). Xiaowei Tang, academician of Chinese Academy of Sciences and a highly esteemed physicist and interdisciplinary scientist, asserts that a general theory of integration is reasonably able to underwrite the mind–brain relationship and the riddle of consciousness. In *Brain, Mind and Consciousness* (Tang, 2016), Tang argues that a unified theory of psychology and an integrated approach to brain and cognition can be drawn based on the integration of mental interactions (p. 1).

This book divides into 20 chapters across five parts. As a well-disciplined physicist, Tang tends to see brain, mind, and consciousness separately due to integration in various levels of explanations, drawing transferable methodologically micro–macro integration of conception from physics. Thus, in the “Preface,” Tang says the book gives a sketch of five forms of interactions in the mental world: mental components interaction, mind–brain interaction, mind–body interaction, mind–environment interaction, and mind–society interaction (p. 1). Afterwards, he discusses “Brain” (Part I), “Mind” (Part II), “Consciousness” (Part III), “Dreaming” (Part IV), and “Unified Theories of Psychology and Cognition” (Part V), successively.

Part I (Chapters 1–3) focuses on the integration of brain, of which neural architectures are regarded as the most reliable empirical basis of mental integration. Tang proposes a theory dubbed as Four Functional Systems (FFS) (of the brain) according to aspects of the hierarchical neural systems. Historically, FFS is in the prospects of extending Alexander Luria's theory of Three Functional Systems (Luria, 1973). The first subsystem makes preparations for potentiation, brain activation, and awareness states; the second one receives, processes, and stores the incoming sensory information; the third one formulates and regulates subsequent mental activities and behaviors; and the last one evaluates stored information and then, in turn, generates emotional feelings. Thus, the complexity of the brain—as an integrated whole—is identical to the brain's quaternary functional systems.

Part II (Chapters 4–6) centers on the integration of mind. Tang treats unconscious mental activities as the prerequisites of consciousness. Furthermore, associating with FFS, he points out that the mind is involved with four components: arousal, cognition, emotion, and volition (pp. 28–30). These components—the tetrads of *a* mind—are neither operated separately nor combined mechanically. They interact with each other in a self-organizing way.

Part III (Chapters 7–12) showcases Tang's theory of consciousness. In Chapter 7, correspondingly, he proposes Four Components of Consciousness (FCC). Consciousness is composed of four elements: awareness, content, intention, and emotion (p. 51). Tang tries to link the arousal states of consciousness with the energy states of (human) brain anatomical regions. He believes that the interactions of brain regions fundamentally result in those regions' transferable variable states. In particular, based on Fechner's interpretations of external and internal psychophysics, Tang adopts mathematical and physical methods to illustrate consciousness. In his view, consciousness is not an all-or-nothing phenomenon; the emergence of consciousness has a process. Rigorous formulas can describe this process and the relationship between brain and mind (p. 90–95).

Part IV (Chapters 13–17) discusses dreaming. Studies have shown that most dreams appear during rapid eye movement. Neurotechnologies, such as electroencephalogram, can record the activation and dynamic changes of different brain regions during dreams, indicating that information processing continues. Information processing occurs during both dreaming and waking states, but the same neural basis corresponds to different characteristics. For dreaming is a unique state of consciousness, understanding the nature of dreaming is of great significance to understanding consciousness.

Finally, based on the previous four sections' empirical and theoretical support, Part V (Chapters 18–20) gets ready to construct a unified theory of psychology and cognition. Both psychologists and cognitive scientists aspire to a unified theory of the mind as physicists have expected (simply relatively speaking) in the material realm. Resorting to a general theory of physical and mental integration, Tang plans a Grand Unified Psychology program, whose theoretical framework consists of four parts: (1) central viewpoints and methods of studying psychology and behavioral science, (2) critical examinations of theories in various sub-fields of psychology and somewhat convergent psychological phenomena, (3) essentials of unifying various research fields and disciplines of psychology, and (4) essentials of unifying the research diagram. Tang stresses the significance of studying the mind from mental integration (and psychological interaction). Elsewhere, Tang advocates the following: the processes and phenomena of cognition are only one dimension of the psychological realm; consequently, it is necessary to study different (mental) interactions of psychological experiences and feelings involving cognition, such as understanding, evaluating, and monitoring. In short, the units and correlations of various mental interactions involving cognition and feelings are indispensable.

Throughout the book, Tang attempts to systematically grasp the interdisciplinary advances of physics, psychology, brain science, medicine, and technical science and present his mind and consciousness theory (FFS and FCC). In the end, he offers a sketch of a unified program of psychological and cognitive sciences. This book epitomizes Tang's research on mind, brain, and consciousness over the past two decades and could be seen as the forefront of Chinese scholars' research on consciousness. And in a way the above system that the book contains is so extensive that some fascinating details, such as experimental studies on mind wandering and quantitative studies of consciousness, cannot be specifically presented. Just as Kandel (2018) has stated, “determining the nature of consciousness is one of the greatest scientific challenges of the twenty-first century, so answers will not come quickly or easily,” the theories and program proposed by Tang in this book need to be scrutinized further by contemporaries.

## Author Contributions

JZ and DD wrote the manuscript, with larger contributions by JZ. DD then provided edits and suggestions for revision. Both authors contributed to the article and approved the submitted version.

## Conflict of Interest

The authors declare that the research was conducted in the absence of any commercial or financial relationships that could be construed as a potential conflict of interest.

## References

[B1] Doerig A. Schurger A. Herzog M. H. (2020). Hard criteria for empirical theories of consciousness. Cogn. Neurosci. 12, 41–62. 10.1080/17588928.2020.1772214 32663056

[B2] Gennaro R. J. (ed.). (2018). The Routledge Handbook of Consciousness. New York, NY; London: Routledge.

[B3] Kandel E. R. (2018). The Disordered Mind: What Unusual Brains Tell Us About Ourselves. New York, NY: Farrar, Straus and Giroux.

[B4] Luria A. (1973). The Working Brain: An Introduction to Neuropsychology. New York, NY: Basic Books.

[B5] Tang X. (2016). Brain, Mind, and Consciousness. Hangzhou: Zhejiang University Press.

